# Music on Prescription to Aid Sleep Quality: A Literature Review

**DOI:** 10.3389/fpsyg.2020.01695

**Published:** 2020-07-28

**Authors:** Gaelen Thomas Dickson, Emery Schubert

**Affiliations:** Empirical Musicology Laboratory, University of New South Wales, Sydney, NSW, Australia

**Keywords:** sleep, music, therapy, insomnia, conditioning, arts on prescription

## Abstract

Research on the efficacy of music for improving sleep quality has produced mixed results. We investigated whether the number of music dosages could be a reason for the lack of clarity. Six longitudinal music sleep studies using the Pittsburgh Sleep Quality Index (PSQI) over 3 weeks were identified. Music when compared to active (audiobooks or medication) or passive controls significantly improved (improvement is reflected by a lower score) PSQI within the first or second week of prescription. The improvement was an average mean difference of −1.15 (*SD* = 0.53) for each week. Music dosages continued to be associated with improved PSQI over a study that had a 3-month music intervention. One study with a low initial PSQI score resulted in poor sleepers (PSQI > 5) achieving healthy sleep (PSQI < 5) within 3 weeks of regular music intervention. For future studies, “prescribing” music beyond 3 weeks may lead to more instances of healthy sleep, particularly for those who have mild sleep problems. To explain the findings, we proposed that the relationship between weeks of music listening and improved PSQI are attributed to the truncation of poor bedtime habits linked to ruminative tendencies and consequent hyperarousal prior to the music intervention. Music listening at bedtime replaces those bad habits, we argue, by forming a new psychological link between bedtime and sleep through evaluative conditioning. The findings of the present study provide disarming evidence of the potential for prescription of music for treating mild sleep disorder.

There is a growing body of research supporting music as an effective non-pharmacological sleep aid (De Niet et al., 2009b; Wang et al., 2014; Jespersen et al., 2015; Feng et al., 2018). For example, a meta-analysis of 17 non-pharmacological sleep aids found music-assisted relaxation to be the only intervention with a moderate effect size (De Niet et al., 2009a). However, there are exceptions. Lazic and Ogilvie (2007) found that music did not improve sleep quality after a single exposure to music at bedtime. On the other hand, Chan et al. (2010) suggested that at least 4 weeks of treatment is required to reveal the effectiveness of music as a sleep aid. The regularity of bedtime music interventions may, therefore, have some impact on the efficacy of music for improving sleep. Assessment of sleep quality at several time points separated by a week is central to good diagnostic practice (Hoch and Reynolds, 1986; Buysse et al., 1989). Assessment of the influence of increased exposure to music as a sleep aid is therefore needed.

This connects seamlessly with the Arts on Prescription movement, which is a complementary approach to improving health and well-being where a creative activity is prescribed to individuals over a period of time (Bungay and Clift, 2010; Jensen et al., 2017). The prescription of music as “treatment” has been explored in the context of “music as a coping strategy” with a focus on improving physiological and psychological well-being (Jacobsen et al., 2018). Jacobsen et al. (2018) suggested that identifying the quantity or “dose” is a challenge for future research in music on prescription. This review, therefore, investigates music for improving sleep using an Arts on Prescription rationale and, in particular, examining the frequency of doses of music required for improving sleep.

## Methods

**FIGURE 1:**
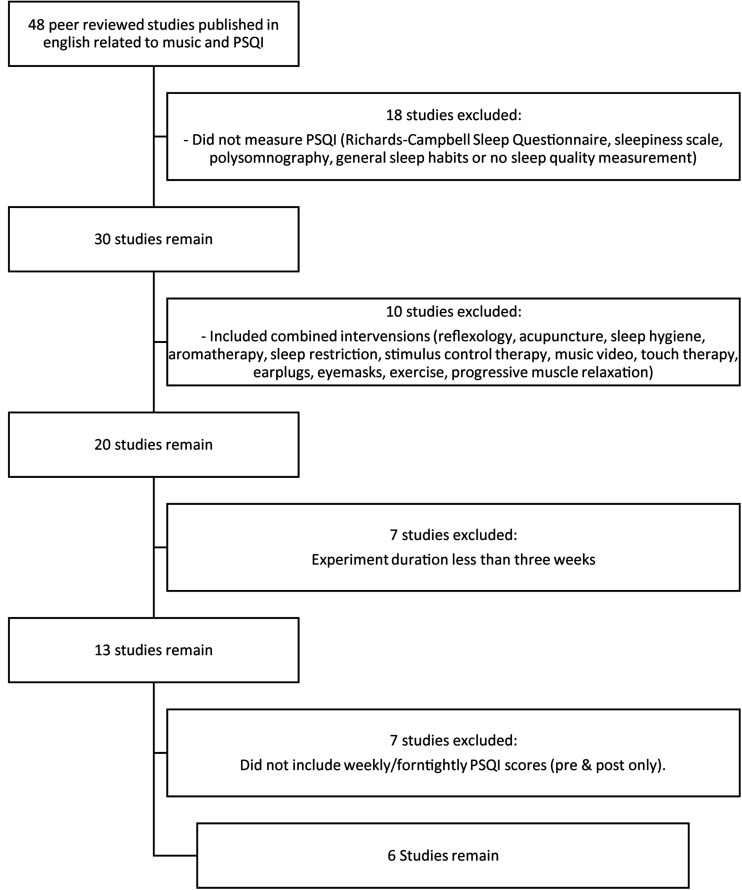
Flowchart of selection of reviewed literature.

The method for investigating the research question was a review of the literature. The inclusion criteria for the review were (1) peer-reviewed research in which music was exclusively played at bedtime sleep and sleep quality was then assessed; (2) in addition to a music intervention, a control condition was reported; (3) a minimum of 3 weeks observation of music as a sleep intervention was applied; (4) weekly or fortnightly reporting of the Pittsburgh Sleep Quality Index (PSQI) was reported for consistency. PSQI is a self-report global scoring of summed components of sleep quality (e.g., sleep onset latency, nighttime waking behaviors, etc.). PSQI scores range from 0 to 21 with lower global scores indicating good sleep quality and global scores above 5 indicating poor sleep (Buysse et al., 1989; Buysse et al., 1991; Tsai et al., 2005). The studies were limited to PSQI because it is the most used measure of sleep quality in music-related studies and so allows for comparisons across studies. Studies that combined interventions with music (such as progressive muscle relaxation or use of eye masks) were excluded. Sample characteristics, music selection, and experimental design were not limited by the inclusion criteria. Papers were identified in online databases PubMed, ScienceDirect, Cochrane Library, and Google Scholar. The search terms used were “PSQI” and “music” with additional papers identified through references and citations. Forty eight papers were identified measuring the impact of music on sleep as measured by PSQI. Of these studies, six papers reported weekly PSQI measurements over 3 weeks (Figure 1).

## Results

**FIGURE 2:**
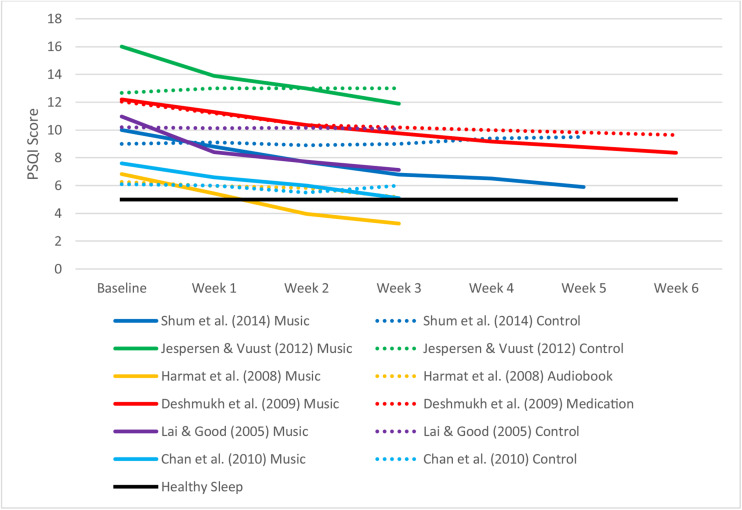
Pittsburgh sleep quality index (PSQI) score for music interventions by exposure.

**FIGURE 3:**
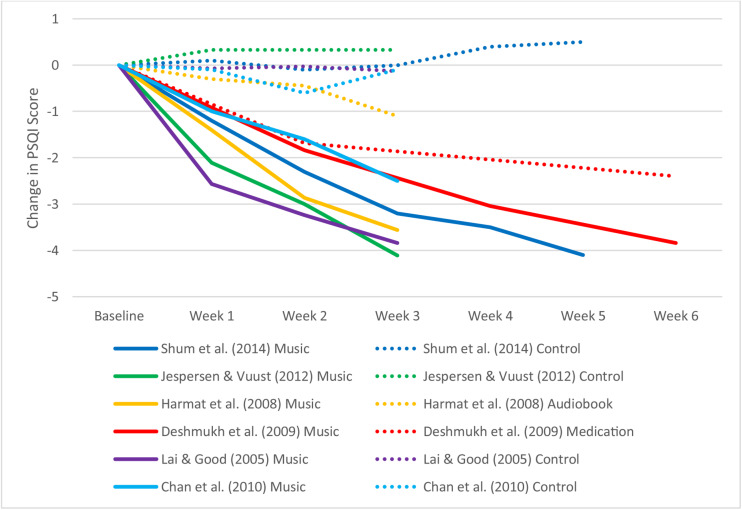
The difference from baseline Pittsburgh sleep quality index (PSQI) scores for music interventions by time.

Table 1 summarizes the characteristics of the included studies. Table 2 and Figure 2 display weekly global PSQI scores for music and control conditions across all included studies. Figure 3 shows the weekly improvement in sleep quality for music conditions in all studies relative to control conditions. Most studies reached statistical significance in week 1 or week 2 (Table 2). Mean change in PSQI was calculated for each week across all studies with the exclusion of Deshmukh et al. (2009) due to its fortnightly measurement. Mean global PSQI decreased in the music intervention by −1.15 (*SD* = 0.538) each week for music conditions, however, the first week had a slightly larger mean difference of −1.66 (*SD* = 0.59), suggesting diminishing improvements or regression to the mean. Global PSQI decreased in the control groups on average by −0.06 each week with effectiveness depending on the type of control (i.e., passive control, audiobook, or medication).

**TABLE 1:** Pittsburgh sleep quality index (PSQI) scores by group at each week.

Source	Group	*n*	Baseline: Mean (*SD*)	Week 1: Mean (*SD*)	Week 2: Mean (*SD*)	Week 3: Mean (*SD*)	Week 4: Mean (*SD*)	Week 5: Mean (*SD*)	Week 6: Mean (*SD*)
Shum et al. (2014)	Music	28	10.0 (2.5)	8.8 (2.5)	7.7 (2.7)	6.8 (2.9)	6.5 (3.1)	5.9 (2.4)	
	Control^a^	32	9.0 (2.4)	9.1 (2.9)	8.9 (2.8)	9.0 (2.6)	9.4 (2.5)	9.5 (2.6)	
Jespersen and Vuust (2012)	Music	9	16.00 (1.41)	13.89 (1.96)	13 (2.57)	11.89 (2.47)			
	Control^a^	6	12.67 (1.86)	13 (1.37)	13 (4.27)	13 (3.10)			
Harmat et al. (2008)	Music	35	6.83 (2.09)	5.43 (2.42)	3.97 (2.13)	3.27 (1.80)			
	Audiobook	30	6.27 (1.72)	5.97 (2.06)	5.83 (2.52)	5.17 (2.21)			
Deshmukh et al. (2009)	Music	23	12.20 (2.02)		10.36 (2.23)		9.16 (2.44)		8.36 (2.69)
	Medication	21	12.04 (2.07)		10.36 (2.06)		10.00 (1.98)		9.64 (2.06)
Lai and Good (2005)	Music	30	10.97 (2.61)	8.40 (3.07)	7.73 (3.15)	7.13 (3.19)			
	Control^a^	30	10.20 (2.82)	10.13 (2.78)	10.17 (2.73)	10.07 (2.75)			
Chan et al. (2010)	Music	21	6.1 (3.7)	6.0 (3.0)	5.5 (3.3)	6.0 (3.6)			
	Control^a^	21	7.6 (4.0)	6.6 (3.2)	6.0 (5.0)	5.1 (2.6)			

*^a^Passive control group (no interventions).*

**TABLE 2:** Music sleep studies and Influence of Pittsburgh Sleep Quality Index (PSQI) over time on the music sleep studies.

Source	Baseline PSQI	PSQI at week 3	Weeks until statistical significance is reached
Shum et al. (2014)	10	6.8	Week 1 (*p* = 0.018)
Jespersen and Vuust (2012)	16	11.89	Week 1 (*p* < 0.021)
Harmat et al. (2008)	6.27	3.27^b^	Week 2 (*p* = 0.0002)
Deshmukh et al. (2009)	12.2	Approx. 9.66	Week 2 (*p* < 0.017)^a^
Lai and Good (2005)	10.97	7.13	Week 1 (*p* < 0.01)
Chan et al. (2010)	7.60	5.1	n/a^c^

*^a^Only included a fortnightly sample (averaged). ^b^PSQI < 5 indicating healthy sleep. ^c^P-value not reported in the study except at week 3.*

Participants receiving music intervention in Harmat et al. (2008) decreased the mean global PSQI score to below 5. This indicates that the participants were no longer poor sleepers (PSQI < 5) at the end of the study. Chan et al. (2010) approached good sleeper outcomes with mean PSQI of 5.1. These lower scores may be explained by the low baseline global PSQI scores for both studies. From an inferential statistical perspective, none of the control groups achieved healthy sleep (PSQI < 5), and only medication in Deshmukh et al. (2009) achieved statistically significant improvements (*p* < 0.05) in PSQI. A longitudinal study by Wang et al. (2016) measured PSQI monthly over a 3-month music intervention period. They reported global PSQI of 13.53 at baseline, 9.28 at 1 month, 8.28 at 2 months, and 7.28 at 3 months. This equates to a decrease in PSQI of −3.87 at month 1, −1 at month 2, and −1 at month 3. These findings provide further evidence that sustained use of music continues to improve sleep quality over time but with a diminishing effect.

Table 3 reveals the musical characteristics and methodology across the studies investigated. The sample populations included the elderly, students with sleeping complaints, refugees with posttraumatic stress syndrome, and patients suffering from depression. The studies varied in musical genres and music selection processes—with studies reporting use of western classical, Chinese classical, new age, jazz, and raagas. The duration of treatment ranged from 30 minutes across three exposures for 3 weeks (Chan et al., 2010) to music for 60 minutes with 21 exposures for 3 weeks (Jespersen and Vuust, 2012).

**TABLE 3:** Music sleep study characteristics and methods.

Source	Participants	Intervention/treatment	Genre	Features of music	Selection	Schedule	Duration of each treatment	Number of exposures
Shum et al. (2014)	Older community-dwelling adults	Music No treatment	Western Classical, Chinese Classical, New Age, and Jazz	Soft volume Instrumental 60–80 BPM	Participant selected from a list	Not specified	40 min	35
Jespersen and Vuust (2012)	Posttraumatic Stress Disorder in Refugees	Music and pillow Pillow only	New Age	Classical instrumentation Nature sounds 52 BPM Stable dynamic contour Repetitive and simple structure	Prescribed to participants	At bedtime	60 min	21
Harmat et al. (2008)	Students with sleep complaints	Music Audiobook No treatment	Classical	Not specified	Prescribed to participants	At bedtime	45 min	21
Deshmukh et al. (2009)	Depressed patients	Music Antidepressants	Raagas	Flute-based compositions	Prescribed to participants	At bedtime	45 min	45
Lai and Good (2005)	Older community-dwelling adults	Music No treatment	New Age, Eclectic, Popular Oldies, Classical, Slow Jazz, Chinese Folk Music	60–80 BPM No accented beats No percussive characteristics No syncopation	Participant selected from a list	At bedtime	45 min	21
Chan et al. (2010)	Elderly people	Music No treatment	Western Classical, Chinese Classical, Meditative, and Jazz	Flowing Instrumental 60–80 BPM	Participant selected from a list	Not specified	30 min	3

*BPM, beats per minute.*

## Discussion

The findings of the present study provide evidence that sustained music intervention improves sleep quality. To date, no research has proposed reasons for how increasing weeks of exposure to (i.e., frequency of) music at bedtime influences sleep quality. For example, Dickson and Schubert (2019) proposed six reasons why music improves sleep without considering the influence of sustained, regular music dosage. Investigating the reasons why individuals use music as a sleep aid, Trahan et al. (2018) identified the reason “habit.” Some participants stated they listened to music as a sleep aid due to habit or routine. Our study supports this interpretation. Increased frequency of music at bedtime may lead to the habit-forming of music listening as part of a regular sleep routine. Allen et al. (2016) recognized the importance of introducing brief sleep routines for improving sleep outcomes in a review of pediatric studies. They highlighted the importance of bedtime routines being calming activities and being consistent. Music may help to facilitate the psychological aspects of the routine proposed by Allen et al. (2016).

From a psychological perspective, the habit formation that arises by pairing music with good sleep quality (particularly reducing sleep-inhibiting anxiety) is analogous to “evaluative conditioning” (Martin and Levey, 1978), which has been used to explain links between emotion and music (Juslin, 2013). In our explanation, evaluative conditioning involves music [conditioned stimulus (CS)] being repeatedly paired with going to sleep [unconditioned stimulus (US)] until the music evokes the healthy sleep routine, terminating the previously unhealthy sleep routine that was associated with sleep-inhibiting thought patterns. Music (CS) is repeatedly paired with sleep (US) until a strong association is developed between music and sleep; hence, music becomes a sleep-inducing stimulus after several dosages.

In a meta-analysis by Hofmann et al. (2010), the evaluative condition became stronger when the number of CS–US pairings increased. This relationship between CS–US co-occurrences may explain the increase of music and sleep co-occurrences aiding sleep. A case study by Poser et al. (1965) conditioned relaxation to a metronome beat at bedtime for a participant suffering anxiety and insomnia. Poser et al. (1965) achieved this by pairing the metronome beat with anesthetics (methohexitone injections) over 16 trials and later the relaxation conditioning occurred from the metronome without the anesthetics. The participant reported being able to fall asleep successfully after listening to the metronome each night. Because our explanation is dependent on truncating existing habits and replacing them with new ones through the addition of music to the bedtime routine, rather than through a pharmacological UC, more exposures may be needed but has the advantage of averting the need for drug injections and the possible consequent side effects.

The Hyperarousal Model of Insomnia (HMI) stems from the cognitive–behavioral datum that some individuals suffering from insomnia may have learned counterproductive sleep associations from ruminating on insomnia (Riemann et al., 2010). A co-occurrence of anxiety-inducing wakeful thoughts and the bedroom creates an association between the bedroom/bedtime (CS) with anxious/arousing wakeful thoughts (US) instead of sleep. In a review of the HMI, Bonnet and Arand (2010) suggested the treatment of hyperarousal as a remedy for insomnia by finding ways to decrease or normalize inappropriate psychological arousal. Johnson (2003) reduced hyperarousal in elderly women suffering from insomnia. The study measured participants who self-reported issues with sleep through the Stanford Sleepiness Scale for 10 nights without and then with music. The participants rated an increased level of sleepiness at bedtime and decreased sleep onset latency and number of nighttime awakenings when listening to music compared to without. After day 20, each participant was interviewed regarding the experience. The interviews revealed that the elderly women suffered from a great deal of frustration and dread associated with insomnia which was decreased in the second half of the experiment with music. The music may have undone the learned counterproductive sleep associations attributed to rumination over insomnia, thus decreasing hyperarousal. Music created new associations with sleep, but the formation of the association may take time to become established.

All studies included in this review showed improved sleep quality with increased exposure despite differences in sample, music genre and selection process, duration of treatment, and exposure frequency. The continued improvement in sleep quality with increased exposure to music, therefore, appears to be unanimous regardless of context. Only two studies achieved or neared good sleeper outcomes with a PSQI below 5 (Harmat et al., 2008; Chan et al., 2010), both of which may be explained by lower baseline global PSQI scores. Initial PSQI for the four other studies was relatively higher, and thus music interventions continuing for additional weeks beyond those reported may have further led to better sleep (with PSQI eventually falling below five) as demonstrated by Wang et al. (2016).

Another key finding of the present study is that an active control, such as an audiobook, which itself may be viewed as a kind of art intervention, still does not have the same positive impact on sleep as does music. Two studies (Harmat et al., 2008; Jespersen et al., 2019) compared the influence of music to audiobooks on sleep quality with 3 weeks of intervention (exposure). Both studies found that music improved PSQI where the audiobook did not, with well-selected active controls because those were also based on audio stimuli. Harmat et al. (2008) suggested that music may have other features besides evoking relaxation and encouraging expectation for improved sleep which aided sleep. If audiobooks were unable to improve sleep quality where music was able to, perhaps evaluative conditioning is more complicated than any co-occurrence of stimuli at bedtime. Specific features of music enable the co-occurrence of music and sleep to aid sleep. It therefore seems possible that there are some peculiarly mysterious effects of music that science has yet to address.

## Conclusion

This study reviewed the influence of multiple weeks of exposure to music at bedtime upon sleep quality. A literature review compiled longitudinal studies of sleep quality when music interventions were applied. PSQI was used as the measure of sleep quality. We found that music improved global PSQI at a rate of -1.15 for each week of exposure. The rate of benefit declines over time, but in general, sleep quality is not deteriorated by sustained music intervention. Music achieved statistically significant improvements on PSQI by the first or second week. Only studies with already low baseline PSQI scores (that is, only mildly poor sleepers) achieved healthy sleep within 3 weeks, suggesting that music intervention may be most effective for individuals suffering from mild sleep problems.

Continuing music exposure beyond 3 weeks may continue to improve sleep quality as demonstrated through a study conducted over 3 months. The reasons why increased weeks of exposure improved sleep quality were explained through physiological and psychological factors, including evaluative conditioning and a reduction of negative ruminations moderating the individual’s level of hyperarousal. This new exposure-based mechanism of “habit formation” appears to be additional to the researcher-proposed reasons for how music improves sleep identified by Dickson and Schubert (2019). Future research should consider increasing weeks of exposure beyond 3 weeks while applying an active, art-based, audio control (such as audiobooks) to further test our conclusions and to see if healthy sleep can be achieved consistently over a prolonged period.

## Limitations and Future Directions

This study chose to focus on the influence of dosage (exposure) on sleep using PSQI, a general, multidimensional measure of sleep quality. The methodology is limited because it does not pinpoint diverse and specific causes of sleep problems. Jespersen et al. (2019) suggested that the type of sleep problem (initiation insomnia or maintenance insomnia), severity and persistence of sleep problem (subclinical or clinical insomnia), experimental designs with a risk of bias, sample sizes, and other factors can complicate the legitimacy of study outcomes.

This study chose to focus on PSQI because this measure is common throughout music and sleep literature, allowing for comparisons. Future studies should consider comparing the influence of inadequate exposure to music using other tools, including physiological measures (i.e., polysomnography).

Another limitation of the present study is that all papers reviewed used randomization to separate participants into the test intervention and control, instead of groups being matched. This results in some papers having initial PSQI scores that were statistically significantly different at baseline (Jespersen and Vuust, 2012), while another paper did not include comparisons of pretest global PSQI (Shum et al., 2014). Nevertheless, the consistent improvements across the studies investigated suggest a role for continuing dosages of music for mild sleep disorders and provides an intriguing example of the possible legitimacy of Arts on Prescription.

## Author Contributions

GD conceived of the presented idea, developed theory, and performed analytic calculations. ES verified analytical methods and supervised the findings. Both authors discussed the results, wrote the manuscript, and approved the submitted version.

## Conflict of Interest

GD has created the Android and iPhone app “Can’t Sleep”. The remaining author declares that the research was conducted in the absence of any commercial or financial relationships that could be construed as a potential conflict of interest.
